# Participants’ perspectives on mindfulness-based cognitive therapy for inflammatory bowel disease: a qualitative study nested within a pilot randomised controlled trial

**DOI:** 10.1186/s40814-015-0041-z

**Published:** 2016-01-19

**Authors:** Mariyana Schoultz, Leah Macaden, Gill Hubbard

**Affiliations:** School of Health Sciences, University of Stirling, Highland Campus, Inverness, UK

**Keywords:** Inflammatory bowel disease, Mindfulness, MBCT, Focus groups, Qualitative study

## Abstract

**Background:**

Mindfulness-based interventions have shown to improve depression and anxiety symptoms as well as quality of life in patients with inflammatory bowel disease (IBD). However, little is known about the experiences of this group of patients participating in mindfulness interventions. This paper sets out to explore the perspectives of patients with IBD recruited to a pilot randomised controlled trial (RCT) of mindfulness-based cognitive therapy (MBCT) about the intervention.

**Methods:**

In a qualitative study nested within a parallel two-arm pilot RCT of mindfulness-based cognitive therapy for patients with IBD, two focus group interviews (using the same schedule) and a free text postal survey were conducted. Data from both were analysed using thematic analysis. Data and investigator triangulation was performed to enhance confidence in the ensuing findings.

Forty-four patients with IBD were recruited to the pilot RCT from gastroenterology outpatient clinics from two Scottish NHS boards. Eighteen of these patients (ten from mindfulness intervention and eight from control group) also completed a postal survey and participated in two focus groups after completing post intervention assessments.

**Results:**

The major themes that emerged from the data were the following: perceived benefits of MBCT for IBD, barriers to attending MBCT and expectations about MBCT. Participants identified MBCT as a therapeutic, educational and an inclusive process as key benefits of the intervention. Key barriers included time and travel constraints.

**Conclusions:**

This qualitative study has demonstrated the acceptability of MBCT in a group of patients with IBD. Participants saw MBCT as a therapeutic and educational initiative that transformed their relationship with the illness. The inclusive process and shared experience of MBCT alleviated the sense of social isolation commonly associated with IBD. However, time commitment and travel were recognised as a barrier to MBCT which could potentially influence the degree of therapeutic gain from MBCT for some participants.

**Electronic supplementary material:**

The online version of this article (doi:10.1186/s40814-015-0041-z) contains supplementary material, which is available to authorized users.

## Background

Inflammatory bowel disease (IBD) is a group of chronic gastrointestinal diseases with relapsing nature. The disease affects around 250,000 patients in the UK and around 28 million people worldwide [1, 2]. The two main types are Crohn’s disease (CD) and ulcerative colitis (UC) [3]. IBD incidence and prevalence are increasing with future prevalence likely to be considerably greater than at present [4]. To date, there is no cure for the disease and the main focus is on maintaining remission and keeping relapse at bay, managed primarily by medications [5]. In addition, patients with IBD are at increased risk of surgery and other complications.

The symptoms experienced by both CD and UC are very similar and very distressing. They include and are not exclusive to acute abdominal pain and gut spasms, vomiting, malnutrition, fever, fatigue, diarrhoea and rectal bleeding. These symptoms are potentially disabling and can cause severe impact on both physical and psychosocial wellbeing [6]. In fact, around 30 % of these patients suffer from moderate to severe psychological distress and have difficulties in coping with their illness [7, 8]. This distress is not only because they have to deal with the symptoms described above, but because they often experience fear and humiliation as a result of faecal incontinence, which has a profound effect on family, work, social life and identity [9–11]. In addition, they have to deal with the sense of loss of control, feeling ‘dirty and smelly’ and feeling they are unable to fulfil their potential at work and in sexual relationships [12, 13]. Moreover, the steroidal treatments can often induce side effects such as weight gain and mood swings [14], which might require treatment with antidepressants. Consequently, the proportion of anxiety and depression among these patients is not related to the disease severity, but to their level of perceived stress [15, 16].

There is a considerable body of research investigating the relationship between perceived stress and mindfulness. A number of studies have reported a negative correlation between perceived stress and mindfulness [17–19]. Mindfulness or mindful awareness is the core skill taught in mindfulness-based interventions such as mindfulness-based stress reduction (MBSR) and mindfulness-based cognitive therapy (MBCT). MBCT combines cognitive therapy with MBSR (a programme developed by Jon Kabat-Zinn initially for patient with chronic medical conditions) [20]. MBCT is an 8-week, facilitator-led, group-based psychological intervention. In addition to the 2-h weekly group sessions, there is a guided home practice component for up to 45 min a day. Within the 8 weeks, participants practice a series of mindfulness meditation, cognitive behavioural therapy and stretching exercises within the group and at home [21]. During the programme, participants become more aware of their body sensations, thoughts and emotions and their interrelatedness [21]. This process of cultivating awareness enables participants to explore the change (shift) in their perception and learn how to recognise certain maladaptive thinking patterns and behaviours and replace them with more helpful ones [22]. The National Institute for Health and Care Excellence (NICE) guidance recommends MBCT as a preferred psychological therapy for recurrent depression and chronic worry [23].

Previous studies have demonstrated effectiveness of MBCT for patients with depression and anxiety [24], chronic pain [25], fibromyalgia [26] and chronic fatigue syndrome [27]. In addition, there is an encouraging early evidence about the use of mindfulness-based interventions in either patients with CD or UC in improving depression and anxiety scores and quality of life [28, 29]. However, little is known about the perceptions of this group of patients about any perceived therapeutic benefit of mindfulness-based interventions and particularly MBCT. Thus, this embedded qualitative study is the first of its kind in its investigation and is aiming to close such an evidence gap.

This paper reports the findings from two focus groups and free text survey. This nested study together with a pilot RCT is part of a wider developmental and feasibility work for a full-scale RCT testing if MBCT can be used in IBD patient care as an adjunct therapy. This is in line with the Medical Research Council (MRC) framework for development and evaluation of complex interventions [30], which recommends that such a process evaluation, nested within a trial, can provide an insight into why an intervention works and how it can be optimised for patient benefit.

Nested design is when one methodology (for example qualitative) is placed within the framework of another (for example quantitative) [31].The pilot RCT, where this qualitative study was nested, was a parallel two-arm, exploratory pilot RCT (MBCT treatment, *n* = 22 versus wait-list control group, *n* = 22) with three assessments (baseline, post-treatment and 6-month follow-up). The full protocol of the pilot RCT and its results are reported elsewhere [32, 33].

Although the nested RCT designs across the health and social sciences are rising [34], there is still some criticism over using this design [35]. The criticism is mainly due to researchers not having sufficient and adequate guidance of how to deal with issues intrinsic to nesting qualitative methods within an RCT framework [36]. Recently, such guidance has been developed by O’Cathain et al. [37] for maximising the impact of qualitative research in feasibility and pilot randomised controlled studies (RCT). This qualitative study fits with the above guidance, and the focus groups and survey from the nested qualitative study were included as an important element of the pilot study to provide a better understanding on how acceptable the MBCT programme is for this group of patients.

### Objectives

This qualitative study aimed to explore the perspectives of patients with IBD recruited to a pilot RCT of MBCT about the intervention.

## Methods

The consolidated criteria for reporting qualitative research (COREQ), a 32-item checklist (see Table 1) for interviews and focus groups [38], was used to guide the structure of this paper.

**Table 1:** COREQ 32 checklist

No	Item	Guide questions/description	Response
Domain 1: Research team and reflexivity			
Personal characteristics			
1.	Interviewer/facilitator	Which author/s conducted the interview or focus group?	Mariyana Schoultz Clinical Academic Fellow at University of Stirling PhD Candidate (conducted focus groups).
2.	Credentials	What were the researcher’s credentials? e.g. PhD, MD	Leah Macaden Lecturer University of Stirling PhD.
3.	Occupation	What was their occupation at the time of the study?	Gill Hubbard Reader University of Stirling PhD.
4.	Gender	Was the researcher male or female?	All 3 researchers are female.
5.	Experience and training	What experience or training did the researcher have?	MS had a qualitative research training through the PhD and have previously conducted qualitative research.
			LM and GH are both qualitative researchers.
Relationship with participants			
6.	Relationship established	Was a relationship established prior to study commencement?	The participants were not acquainted to the researchers prior to the study commencements.
7.	Participant knowledge of the interviewer	What did the participants know about the researcher? e.g. personal goals, reasons for doing the research	The participants knew that the intent of the evaluation was to identify benefits and barriers encountered in order to make improvements to the MBCT programme. Interviewees knew that the researchers were affiliated with University of Stirling.
8.	Interviewer characteristics	What characteristics were reported about the interviewer/facilitator? e.g. Bias, assumptions, reasons and interests in the research topic	
Domain 2: Study design			
Theoretical framework			
9.	Methodological orientation and theory	What methodological orientation was stated to underpin the study? e.g. grounded theory, discourse analysis, ethnography, phenomenology, content analysis	We used a thematic analysis approach (see [45]).
Participant selection			
10.	Sampling	How were participants selected? e.g. purposive, convenience, consecutive, snowball	Participants were recruited consecutively.
11.	Method of approach	How were participants approached? e.g. face-to-face, telephone, mail, email	Participants were approached by mail and recruited by face-to-face.
12.	Sample size	How many participants were in the study?	18 in total.
13.	Non-participation	How many people refused to participate or dropped out? Reasons?	6 participants that were invited for the focus groups did not respond.
Setting			
14.	Setting of data collection	Where was the data collected? e*.*g. home, clinic, workplace	Focus groups were conducted at University Building.
15.	Presence of non-participants	Was anyone else present besides the participants and researchers?	No.
16.	Description of sample	What are the important characteristics of the sample? e.g. demographic data, date	Age, gender, level of education, income, marital status and disease type were included in Table 2 reported in Table 4 of the manuscript.
Data collection			
17.	Interview guide	Were questions, prompts, guides provided by the authors? Was it pilot tested?	Prompts and questions were provided by the author. The guides were not tested in a pilot study, but were discussed.
18.	Repeat interviews	Were repeat interviews carried out? If yes, how many?	No.
19.	Audio/visual recording	Did the research use audio or visual recording to collect the data?	Focus groups was audio-recorded and transcribed prior to analysis.
20.	Field notes	Were field notes made during and/or after the interview or focus group?	No.
21.	Duration	What was the duration of the interviews or focus group?	Focus groups: 1 hour approx.
22.	Data saturation	Was data saturation discussed?	Yes—data collection from participants ended when saturation was achieved.
23.	Transcripts returned	Were transcripts returned to participants for comment and/or correction?	No. Transcripts were reviewed by researchers who listened to the audio recordings to verify their accuracy.
Domain 3: Analysis and findings			
Data analysis			
24.	Number of data coders	How many data coders coded the data?	2 researchers (MS and LM).
25.	Description of the coding tree	Did authors provide a description of the coding tree?	No. However, initial coding was informed by the interview guides but codes were continually refined as simultaneous data collection & analysis provided new insights. Codes were grouped into similar descriptive categories. The final themes were agreed upon by the analysis team through consensus.
26.	Derivation of themes	Were themes identified in advance or derived from the data?	
27.	Software	What software, if applicable, was used to manage the data?	No software was used.
28.	Participant checking	Did participants provide feedback on the findings?	No.
Reporting			
29.	Quotations presented	Were participant quotations presented to illustrate the themes/findings? Was each quotation identified? e.g. participant number	Yes.
30.	Data and findings consistent	Was there consistency between the data presented and the findings?	Yes.
31.	Clarity of major themes	Were major themes clearly presented in the findings?	Yes.
32.	Clarity of minor themes	Is there a description of diverse cases or discussion of minor themes?	Yes.

### Participants and setting

All patients that participated in the qualitative study had consented at baseline to participate in both parts: the pilot RCT and the qualitative study. All participants were recruited from two Scottish NHS boards covering city and rural areas in the north of Scotland. Recruitment took place at outpatient gastroenterology clinics through letter of invitation or in person while attending outpatient clinic. Potential participants were given details of the studies and invited to participate in both parts. At recruitment point, all participants had symptoms in remission. Participants were eligible to participate in the qualitative study if they had completed post intervention assessments. Invitation to the eligible participants with details about the focus groups was made by letter or telephone (depending on their preference).

### Design

As mentioned above, this qualitative study was nested within a parallel two-arm pilot RCT of MBCT for patients with IBD. The pilot RCT had three assessment points: at baseline, post intervention and 6-month follow-up. All participants who completed the post intervention assessments from both arms were invited to participate in the focus groups as well as to complete a free text postal survey. Further details about the full design of the pilot RCT is published elsewhere [32, 33].

### Data collection

Qualitative data was gathered through two focus groups and a postal survey involving patients with IBD participating in a pilot randomised controlled trial exploring the use of MBCT. The focus groups were audio-recorded and took place at a university campus.

### Focus groups

The focus groups aimed to explore and understand the views and experiences the participating group of patients had about the 8-week MBCT intervention. Both focus groups examined potential benefits and barriers of MBCT. A facilitator familiar with the aims of the qualitative study and the pilot RCT facilitated both focus groups. Two focus groups lasting approximately 60 min each were conducted with each focus group following the same predesigned topic schedule in order to maintain the methodological consistency. The topic schedule was based on the questions used in the free text survey. A full topic schedule is presented in Additional file 1. The schedule reflected the specific acceptability questions set out in the pilot RCT protocol [32] and guided by the MRC guidance for developing and evaluating complex interventions [30] to determine how well the intervention was received by the target population [39]. In addition, a wide literature search on psychological interventions identified a range of issues related to expectations, benefits, barriers, availability and length of programme. The topic guide was piloted with volunteering colleagues. Travel expenses were reimbursed for all participants. The first focus group took place a week after the MBCT follow-up assessments were completed, consisting of *n* = 11 participants (four from the MBCT intervention arm and seven from the control arm). The second focus group took place 3 weeks later and comprised of *n* = 7 participants (six from the MBCT intervention arm one from the control arm). All participants self-selected to participate into the first or the second focus group depending on their availability and convenience and consented to the discussions being audio-recorded.

Focus groups have long been recognised as a purposeful interaction for generating data [40] and were used in this study because they created an opportunity for participants to reflect on their responses, which cannot be captured in a questionnaire [41]. In addition, using focus groups is particularly useful when investigating not only *what* participants think about a topic, but also *why* participants think in that particular way [42].

### Survey

A cross-sectional postal survey was sent to all the participants following completion of post intervention assessments. The survey consisted of a series of questions including free text to give opportunity to participants to express their perceptions about their experiences of the MBCT programme. The questions used in the survey mirrored those of the focus group topic themes (see Additional file 2). Participants then sent the completed and anonymised survey (containing only study ID number) in a self-addressed prepaid envelope back to the researcher. A reminder to complete the survey was sent after 2 weeks.

Survey research is the most common method of collecting data in health and health service research, designed to provide a snapshot of how things are at a specific moment [43]. It is a tool that can be used when describing attitudes, experience and behaviours [44], which fits with this study research question. The survey was used in addition to the focus groups to maximise response from participants about MBCT, particularly from those living remotely and to validate the results from the focus groups.

### Analysis

Two researchers (MS, LM) analysed the data, one of whom was involved in conducting the focus groups. The focus groups were audio-recorded and transcribed verbatim. Respondent validation was undertaken during focus groups to ensure rigour with data collected. Data were analysed using a thematic analysis framework approach [45]. This approach is a rigorous method that provides a structure for qualitative data to be organised and coded and for themes to be identified. Initially, the first three phases of familiarisation with data, generating initial codes and searching for themes among codes were done independently by the two researchers for the survey and the focus groups separately. This is referred to as an investigator triangulation [46]. The researchers then met to discuss their findings for the initial analysis. All initial codes and themes were compared and any differences rectified. The researchers then extracted the core themes and went through the next three phases of reviewing themes, defining and naming themes for the survey and the focus groups. Before producing the final report of key themes and sub themes (see Table 2), triangulation of data was performed between the survey and focus groups. The data and investigator triangulation was performed to enhance confidence in the ensued findings as well as to reduce the uncertainty of interpretation [46, 47].

**Table 2:** Overview of themes and subthemes derived from the focus groups and survey

Main themes	Subthemes
Benefits from MBCT	MBCT as a healing/therapeutic process
	MBCT as an educational process
	MBCT as an inclusive process
Barriers for MBCT	Time
	Travel
Expectations about MBCT	Open
	Fixed

### MBCT intervention

The intervention referred to in this study was MBCT, which followed the 8-week MBCT manual developed by Segal et al. [48]. This included eight 2-h weekly face-to-face group sessions. Each session included a facilitator instruction which included a new theme (see Additional file 3 for themes) and instruction for a new group practice/exercise. This was then followed by reflection of practice and then a review and instruction for home practice. Each session would normally close with sitting meditation. A sample list of activities for session 1 is presented in Additional file 4.

Home practice assignments were the other part of the MBCT intervention. The aim of the home practice is to reinforce the learning of the in-group strategies and techniques. The recommended ‘dose’ of home practice was up to 45 min a day, 6 days a week guided by an audio CD and outlined home practice instructions. The home practice handouts and audio files used for home practice are readily available for the published books respectively [48, 49].

### Research team

Two investigators (MS and LM) were involved in data analysis, with the lead author conducting the focus groups and collecting all the data. None of the investigators were involved in providing patient care, and both investigators were employed by the University of Stirling as a clinical academic fellow and a lecturer. A third researcher (GH), employed as a reader by the university, overlooked the process. All of the researchers had previous experience of qualitative research and thematic analysis, with two of the researchers having extensive experience in thematic analysis.

### Ethics

A favourable ethical approval was granted by the NRES Committee for North of Scotland on 8 April 2013 (REC ref 13/NF/0018). NHS Highland and NHS Grampian R&D Management Approval was obtained on 9 April 2013 and 14 September 2013, respectively.

## Results

### Characteristics of respondents

Out of the 44 patients who consented to participate in the pilot RCT, 24 of them completed follow-up assessments and were eligible to participate in the focus groups and complete the postal survey (see Additional file 5 for patient flow). Six participants declined participation in the focus groups and did not return a completed survey. Thus, this section presents the results for the 18 participants that took part in both, the focus groups and the survey. The focus group and survey participants’ characteristics are shown in Table 3.

**Table 3:** Focus groups and survey demographic and clinical characteristics of participants

Baseline	
Age (years) (*n*, mean (sd))	18, 45 (13.86)
Sex F (*n*, (%)), M (*n*, (%))	13 (72.3), 5 (27.7)
Income (*n*, %)	
Less 10K	4 (22, 2)
10K–19K	3 (16, 6)
20K–29K	3 (16, 6)
30K–39K	5 (27, 7)
40K–50K	2 (11, 1)
50K+	1 (5, 5)
Disease type	
CD (*n*, %)	8 (44)
UC (*n*, %)	10 (66)
Marital status	
Single (*n*, %)	5 (27, 7)
Married/cohabiting (*n*, %)	11 (61, 1)
Widowed (*n*, %)	0 (0)
Separated/divorced (*n*, %)	2 (11, 1)
Education	
High school (*n*, %)	5 (27, 7)
Diploma (*n*, %)	9 (50.0)
Degree or above (*n*, %)	4 (22, 2)
Geographical area	
Rural (*n*, %)	9 (50)
Urban (*n*, %)	9 (50)

The key themes that emerged across both focus groups and the free text from the survey were the following: ‘Benefits of Mindfulness-Based Cognitive Therapy for IBD’, ‘Barriers for Mindfulness-Based Cognitive Therapy for IBD’ and ‘Expectations about Mindfulness-Based Cognitive Therapy’. All of the major themes had few sub themes each (see Table 2) that were interlinked. The foundations for these themes are described separately alongside the verbatim quotes.

### Benefits of mindfulness-based cognitive therapy for IBD patients

Three sub themes emerged from the above theme: (1) healing/therapeutic process, (2) educational process and (3) an inclusive process.

1. Healing/therapeutic process
  The perceived main benefits of MBCT for patients with IBD were that participants saw the MBCT intervention as a healing/therapeutic process. Participants noticed that the intervention offered alternative methods for dealing with anxiety, poor sleep, pain and depression (Table 4, quotes 1, 2 and 3) and insight into gaining more control over the management of their symptoms (Table 4, quote 5). Being able to relax and regain control over symptoms is something that is very important for patients with IBD as dealing with pain and other daily symptoms brings on anxiety, depression and poor sleep for many of them [50]. These are normally managed by medications, which often have side effects and further issues. Being able to learn techniques that will ease the pain, help to relax, feel calmer and have a better sleep, while not having to rely on medications, is something that more than a few described as essential to therapeutic gain.

**Table 4:** Benefits quotes

MBCT as a healing/therapeutic process		
Quote no.	Codes	Quote
1	Easing the pain	‘..it's, it’s mostly painkillers that I’m on — I’ve hardly used any in the last eight weeks, hardly any at all, so em…’ (P014)
2	Antidepressant/de-stress and pain relief	‘The short meditation technique is a great way to control yourself-where anti-depressants can cause bigger issues to the user/stress, helps you to relax and ease the pain away’ (P010)
3	Better sleep, sense of peace	‘I feel calmer, and the brain feels less rushed, em you’ve just got a more inner sense of peace.. I’m, I’m not a poor sleeper, but I was a light sleeper — my quality of sleep’s improved that I can sleep straight through and feel you know that I’ve had a good sleep’ (P015)
4	An antidote for anxiety, worry and depression	‘not worrying about something that's happened, or something that may, you know, and just slowing down really’ (P014)
5	Stress and pain relief	‘Gave me an insight on how I can relax, through breathing techniques and can do it anywhere anytime. Help in working through pain to relax through cramps, sleep. ‘(P001)
6	Relaxation	‘helps me to deal with immediate crisis…allows me to relax for a short period.’ (P008)
7	Helping with spiralling down (depression)	‘I still have that urge just to go to bed, just pull the covers over my head and, not sleep necessarily, but just … curl up in a ball and just lie there…. but now I go up and I just, I go ‘I’m doing meditation, I’ll be half an hour,’ and that’ll gimme the kick in the arse that I need … really, I suppose getting me out of really spiralling downward, eh or maybe that was just habit …’(P012)
8	Help with food	‘I find that food tastes differently because I’m taking time to eat it and taste it …’ (P016)
Educational and transformational process		
9	Learned self-care and change of relationship with illness and self	‘I think the fact that I learnt to think about me for a while and to learn to look after myself and to learn when my body is telling me to relax and give it a rest’ (P002)
10	Realisation about taking charge of wellness	‘I understand now that it is ok to stop for 5-10 minutes & its ok to think about me for a change. The processes involved in this programme made me realize I was quite unwell and have helped me throughout’ (P013)
11	Transformed through reflection	‘I realised I was doing everything at high speed and more than one thing at a time, whether it was at home or at work, I was never switching off … I could do that as I was walking to work, and even at work you know I've seen the benefit now of focusing on what I'm doing — being in the moment’ (P008).
12	Learned acceptance	‘as it opened my eyes to accept the illness, but also the mental and physical exhaustion that comes with it.’(P001)
13	Evaluate, change	‘made me stop & evaluate my life.& realize I was able to take control of my body. No longer on auto pilot.’ (P017)
14	Alternative to getting ‘wound up’	‘You don’t have to go to class or yoga. You can actually do it anywhere at any time when you ready to do your three minute breathing you can just take that time out and that’s.. ammm to allow the anger and other things that get you all wound up ..coz a lot of time I get grrrrrr I get really wound up about over other things and full of frustration of whatever and… it and that has helped me over in a really difficult time. its It’s kind of alternative to get wound up. Aha’ (P008)
	Can do it anywhere	
15	Stress relief tools	‘Help me deal with stress, gave me the tools.’ (P003)
16	Everyday application	‘Now I don’t really like closed spaces, and how tight is it inside that blinking MRI thing?..And when they slid me inside I was just about to squeeze that wee thing and shout ‘get me out of here!’ And you remember on the course we did that — near the end of the course — we did that meditation where we were thinking about being a mountain?..well I used the visualisation technique in that MRI scanner, I started to breath, started to, just try and calm myself down; and I was in that thing for an hour and fifteen minutes with no problems at all’ (P015)
Process of inclusion with emotional support		
17	Supported, not judged	I felt very supported and not judged for feeling emotional and not having to explain myself
18	Belonging	‘It’s almost like…little club …its like a everyone gets to know everybody and you didn’t feel …you know the barriers were coming away and everybody was…. I felt …more freely talking..’(P013)
19	Belonging	‘Being part of a group of fellow sufferers was positive…non-judgemental’ (P017)
20	Non-judgemental environment	‘But that’s what it was all about, allowing us to be, ‘cause I know I was emotional one night and I went away and I was quite relieved when I came back and nobody sort of said “are you all right?,” and I, you know I felt that it, it was comfortable to be like that.. I’ll come back and go with this and be supported, and that’s what it’s been like; nobody’s judging or … and that’s so important …’ (P013)
21	Not alone	‘To know you are not alone is a boost to moral in itself’ (P008)
22	Isolation	‘IBD can feel very alone and isolated’ (P004)
23	Share experiences with others	‘It was reassuring to meet others in a similar situation where you can share experiences which very often turn out to be very similar to your own.’ (P001)
  Many patients with IBD worry about how they would deal with a flair up (symptom exacerbation) if it happens and often become either anxious or depressed about it. Participants found the intervention helpful (both emotionally and on a practical level) in dealing with their worry, anxiety and depressive thoughts. They found the programme particularly useful in changing their unhelpful habitual behaviours such as spiralling downward into depressive thoughts with more helpful ones, but also providing them with a respite from their anxiety and a way of dealing with crisis (Table 4, quotes 4, 6 and 7).
  Moreover, due to the nature of the disease, food or relationship with food is often a challenging aspect. These patients often cannot keep food down due to vomiting which brings a lot of fear and anxiety related to food and eating. This contributes to further malnutrition in addition to the malnutrition caused by inflammation of the gut. Thus, for some, like participant P016, restarting to enjoy eating was a significant therapeutic experience (Table 4, quote 8).
2. Educational process
  Some of the participants saw the intervention as an educational process. There was a prevailing perception of learning to self-care as a result of the intervention as described by participants’ quote (Table 4, quote 9). The learned techniques that facilitated self-care and coping were seen through the awareness and recognition when it was time to slow down or even stop before becoming too unwell (Table 4, quotes 10 and 11). The realisation of being able to take control of their body and relinquishing the state of ‘auto pilot’ transformed the relationship with self and the illness (Table 4, quote 13). For another participant, the transformation was through the learned acceptance of the illness, by timely recognition of mental and physical exhaustion (Table 4, quote 12).
  Participants agreed that the applications of the skills learned through MBCT are transferrable and applicable to different everyday situations. They found that MBCT gave them the tools which can be used in different ways and be applied to everyday living while dealing with cramps, stress and other daily frustrations (Table 4, quotes 14, 5 and 15).
  One noteworthy example of ‘everyday’ application of the mindfulness skills was described by a patient who normally struggled with using the MRI scanner (IBD patients often need scans to check changes in the bowel [51]), but using one of the techniques from the class helped the participant cope with the event better (Table 4, quote 16).
3. Inclusive process
  And finally, some of the participants saw the intervention as an inclusive process that facilitated peer support and a supportive environment that resulted in general feeling of inclusion and less isolation.
  Participants believed that the MBCT intervention for patients with IBD provided them with an opportunity for peer support in an inclusive and supportive environment. Their view was that the peer support ascended from shared experiences of participating together in group-based exercises, shared experience of similar diagnosis and shared experience of learning in an environment where they felt safe to express their emotions without being judged. For example, one participant identified that the sense of support in the group was of a great value for them (Table 4, quote 17). Another participant described the sense of support from the peers as being in a ‘little club’ where you can feel free to be yourself (Table 4, quote 18).
  For many participants, not being judged and being left to be themselves in the group made them feel free and comfortable and increased their sense of inclusion and belonging (Table 4, quotes 19 and 20). Consequently, even though the MBCT programme is not a talking therapy, and some participants say very little, the programme offered an opportunity where participants could tap into support from their peers by just being there. For others, it was a confidence booster or a platform where they felt a part of an inclusive and supportive group in which they were free to express themselves and felt less isolated in their illness because of it (Table 4, quotes 21, 22 and 23).

### Difficulties and barriers for mindfulness-based cognitive therapy

Although participants focused on the benefits largely in their discussion, there was a part of the discussion that explored barriers that they faced in accessing or attending the MBCT programme. Within this theme, the largest focus was on ‘time’ and management of time. While for some participants it was difficult to allocate the time for attending the class after work, for others it was the time required to do the home practice component (homework) of the programme. ‘Homework’ was a component of the programme where participants were encouraged to reinforce the MBCT skills through regular practice. Some of the participants recognised their difficulty in juggling work and family life together with the programme and identified that as a barrier (Table 5, quote 1). Others struggled managing to find time for homework and they recognised the effort it takes to fully participate in the programme (Table 5, quotes 2 and 3).

**Table 5:** Difficulties and barriers quotes

Time as a barrier	
Quote no.	Quote
1	‘It was quite difficult having it from 5.30 to 7.30 because that’s when most people will eat especially when you are coming straight from work ..and It’s difficult to get time off off work for doing something like this but erm…’ (P003)
2	‘..but the homework was hard, .. hard to find the time sometimes … but I do, I really enjoy the support …’ (P017)
3	‘Finding the time to do the homeworks sort from week three and onwards.it got quite(pause).. week three and four was quite intense erm..’ (P003)
4	‘it was difficult to fit in all the homework’ (P007)
5	‘yea…some talking..just..(laugh) but also felt coz…I also felt …erm…its taking ,we were having to find time for ourselves. And….erm…’ (P008)
6	‘I think for most folk the time constraints to do meditation were the big issue weren’t they?’ So I can see where some people are coming from when they say they find doing the meditations difficult if you’re busy, but, it’s literally a case o’ trying to find time, isn’t it?’ (P015)
7	‘I didn’t get that much time to do it at home, not as much… but I just didn’t have the time and personally I don’t feel I was committed enough to it as I should have been’ (P007)
8	‘I found you do have to fully embrace its concepts and the time you invest is well rewarded’ (P008)
9	‘once you realise how to prioritise your day, setting aside time to do the homework practices was straightstrait forward.’ (P014)
Distance as a barrier	
10	‘Distance…some.., I don’t come from that far but others come from I bit further and going home this time of year for the next one with the weather and things.’ (P008)

In contrast to the benefits of MBCT where participants recognised that slowing down and finding time for oneself is an integral part of staying well, they also recognised that the key aspect of the programme of finding time was quite difficult. While some participants viewed time as a barrier, there were others who recognised this barrier but worked towards making time for MBCT practice (Table 5, quotes 4 and 5). Other participants recognised that to get the full benefit of the programme, they somehow had ‘to shift’ the way they perceive time and create or find the time in the day to do the home practice (Table 5, quote 6).

While in discussion about time as a barrier to MBCT and home practice, one participant notably acknowledged a key point that perhaps others did not recognise, and that is the personal commitment to make time (Table 5, quote 7).

When talking about time as a barrier, some participants recognised that finding time for practice was related to the attitudes towards committing time for the practice. They talked about time as an investment into wellbeing, that can be very rewarding and about learning to prioritise daily activities in order to create time for home practice (Table 5, quotes 8 and 9).

In addition to the time barrier, travel and distance were difficulties recognised by some. While there were a number of participants that lived rurally, only a small number of participants (two) acknowledged the travel and distance as a barrier to attending the programme. One participant recognised that it could be difficult to travel a distance for some, especially in the winter periods (Table 5, quote 10).

### Expectations about mindfulness-based cognitive therapy

Literature suggests that clarifying patients’ expectations is associated with engagement and health outcome [52]. When participants were asked what their expectations about MBCT were, there were a range of responses. More so, the type of expectations participants had about the MBCT programme seemed to determine their insight and engagement into the programme, and therefore influence their perception about benefits or barriers with MBCT. When looking at the participants responses about expectations, their responses were either very open with very little expectations or very specific and fixed ones. For example, many participants talked about the importance of being open minded before the programme and the willingness (attitude) to go into it with no fixed expectations, such as curing the illness (Table 6, quotes 1, 3, 4 and 5). One participant described how their expectation changed during the programme, from worrying that the programme will be ‘airy-fairy’ to being pleasantly surprised at the outcome (Table 6, quote 2).

**Table 6:** Expectations about MBCT

Flexible (open minded)	
Quote no.	Quote
1	‘Came with an open mind to see how far this could be taken, if it would make me feel better…and I found it great, it was good’ (P015)
2	‘I worried it was going to be hippy, airy fairy, but was pleasantly surprised’ (P003)
3	‘No expectations, open mind’ (P001)
4	‘I didn’t expect to cure my illness, had an open mind and was pretty good.’ (P016)
5	‘It was important to come with an open mind and be willing to go into it.’ (P002)
Fixed (specific)	
6	‘…to understanding the condition and effects it has on me’ (P013)
7	‘…Alternative therapy to drugs, hoping to understand more of how my body works’ (P012)
8	‘My ulcerative colitis would improve. Lifestyle would improve.’ (P008)
9	‘Expected to talk more about depression…I prefer to be in a group and talk about how I feel.’ (P007)

On the other hand, there were participants that described their expectations related to the MBCT programme as more specific and fixed. For example, few were hoping that the programme would help them understand their condition, how the body works and how that affects them (Table 6, quotes 6 and 7). Another participant described her expectations about the programme as being a ‘cure’ for the condition and provide overall improvement of the condition and lifestyle (Table 6, quote 8). While another participant talked about his specific expectation about the content of the programme, which remained unchanged at the end of the programme (Table 6, quote 9).

## Discussion

The study set out to explore participants’ perceptions of mindfulness-based cognitive therapy for patients with IBD recruited to a pilot RCT. It is important to acknowledge that the study used a standard curriculum of MBCT programme [32] which brought a variety of participants’ experiences and perceptions.

The sub themes of therapeutic and educational process were at the heart of the benefits theme. Participants described their personal experience of the programme as a therapeutic and an educational initiative that has enabled them to transform their relationship with the illness and being empowered to take control over their symptom management. This transformative effect of mindfulness on attitudes and perceptions is documented in different clinical and nonclinical populations before [53, 54] and is a result of learning the key skill of being mindful. Thus, learning to respond mindfully to changing life situations and loss has enabled the participants to see stressful events such as their condition, rather than as defining characteristics of their identity. This is particularly noteworthy for patients with IBD, because some of their symptoms such as faecal incontinence and lack of bowel control could lead to loss of self-worth and exacerbate further stress and depression [55, 56]. In addition, through the programme, participants learned that some of their previous habitual responses to stress and symptoms were not always helpful, and therefore, they attained skills that enabled them to change those patterns into more helpful ones. This fits with a proposed mechanism of mindfulness where participants are able to see things from a completely different perspective as a result of the ‘shift’ in perception that happens in the meditation practice [22]. Both the versatile nature of the skills and their transferability into everyday situations acquired through MBCT were viewed as critical benefits by the participants to continue the use of mindfulness.

The shared experience/peer support sub theme has been previously highlighted in IBD and mindfulness literature. Sharing a similar diagnosis can often create a sense of community for the participants, which could alleviate the sense of social isolation commonly experienced by many patients with chronic conditions, including patients with IBD [57–60]. This is particularly important for patients with IBD, as their condition is hidden and they often appear well to others. However, it was very clear in this study that while the shared diagnosis benefit might have attracted participants to partake in the study, the experience of learning together to manage their symptoms in a non-pharmacological way and in a non-judgmental and supportive environment added to the benefit of peer support role and their motivation to stay in the programme. The participants themselves described the importance and value of the group experience as an aid to the therapeutic process and gain. The process of participants sharing their experience of the meditation journey as well as the process of group cohesion growth noted here is been seen in groups of other supportive expressive therapies in IBD and mindfulness-based therapies in other chronic conditions patients [61, 62]. This is particularly interesting since the format of the programme is mainly experiential rather than sharing and talking.

The largest participant focus in the barrier theme was on time or management of time, particularly with home practice. The other barriers identified were travel and distance. It was noted that there was a contrast between the benefits where participants acknowledged the necessity of finding time to listen to the body and its needs through practice; participants also reported that finding time for practice for some of them was very difficult. What is striking that one of the participants clearly pointed out that the attitude of commitment was an integral part in finding or creating time for practice. This attitude of commitment is at the core of the mindfulness practice teaching itself. According to Jon Kabat-Zin, attitudes involve intention and intention sets the stage for the mindfulness practice. Thus, the attitude is described as “the soil in which you will be cultivating your ability to calm your mind and to relax your body, to concentrate and to see more clearly” [20]. Closely connected to the attitude topic is the theme of expectations. Patient expectations in a healthcare setting are a set of beliefs concerned with the treatment/intervention and the outcomes of the treatment [63]. Expectations about interventions have been seen to be associated with engagement and health outcome [52] and therefore influence the perception about benefits or barriers of the intervention. Open and more positive expectations have been linked to better outcomes [64]. This is linked to the mechanisms involved in producing the placebo effect or patients’ belief about outcome can be a powerful predictor of actual outcome or benefit of the intervention [64]. Patient expectation measures should be incorporated into future trials.

### Strengths and limitations

Qualitative analysis of participants’ perceptions and experiences of MBCT for IBD provided within a pilot RCT is novel and has not been explored before. This study was nested within a parallel two-arm pilot RCT examining the use of MBCT for patients with IBD. The data was obtained from participants with a wide range of age, education and social background and geographical area.

The findings from this qualitative study are consistent with and supplement the quantitative data findings from the pilot RCT [33] which showed that depression and anxiety improved at post intervention and 6-month follow-up from baseline in the intervention arm. The findings of this study extend the findings of the pilot in few ways. First, the perceived benefits of the MBCT intervention that participants experienced and discussed in the qualitative study supplements and warrants the recommendation of the pilot RCT for a future full scale RCT exploring the effectiveness of MBCT in IBD. Second, the findings about barriers about the MBCT intervention should be considered in the recruitment process for a full RCT and might be able to ameliorate the higher than expected drop-out rate from the pilot RCT. And last, the findings from the qualitative study about participants’ expectations should be regarded in a future full RCT: patient expectations should be explored and patient expectations measures should be incorporated into such a trial at baseline and follow-ups. This could improve treatment recommendations for effective methods of fostering positive expectations that can ultimately improve health outcomes.

A limitation is that the study had a small sample. As with any qualitative study, the findings of the study cannot be generalised beyond this group of participants, who are unique in their characteristics. However, although the attained benefits of practice and recognised barriers of MBCT for these participants may not be replicated by every patient with IBD, they are certainly possible. The clinical implications of the findings suggest that MBCT could be an acceptable and beneficial intervention for people with IBD and could help them cope better with the disease and symptom management.

Findings also suggest that this intervention may not be appropriate for everybody. Participants’ reports about their initial expectations of the MBCT programme suggest those to be highly important in influencing their experience of the programme and ultimately the therapeutic gain. Participants with fixed expectations and attitudes may need individual support at the start to fully gain the benefits of the programme and should be considered at a point of recruitment.

Because researchers are not immune to their own assumptions about stress, depression and meditation, participants’ own words and phrases were used to allow the reader to make their own judgments about the trustworthiness of the analysis.

## Conclusions

The findings from this study have provided evidence around patient experiences using MBCT in IBD which has not been explored before. The analysis has given an insight into what is important for patients with IBD after diagnosis and when considering psychological therapies. Participants described their personal experience of MBCT as a therapeutic and an educational initiative which has enabled them to transform their relationship with the illness. As a result, many felt empowered with regaining sense of control over their body and symptom management. In addition, the inclusive process and shared experience of MBCT have alleviated the sense of social isolation commonly associated with IBD.

However, time commitment and distance were recognised as a barrier to MBCT and it could potentially influence the degree of therapeutic gain from MBCT for some participants. This study has implications for policymakers and providers of care for patients with IBD. Providers of care may wish to consider offering an 8-week MBCT programme to patients with IBD to help with symptoms of depression and anxiety.

To conclude, this qualitative study has demonstrated the acceptability of MBCT in a group of patients with IBD. Together with the pilot RCT where this study was nested, both findings form a knowledge base for a future full-scale RCT investigating the effectiveness of MBCT in IBD. This trial is novel in its investigation and will hopefully encourage more research to address the psychological needs of patients with IBD and the use of MBCT.

## Additional files

Additional file 1:
**Topic guide schedule for the facilitator with preamble.** (DOC 24 kb)
Additional file 2:
**Free text survey questions.** (DOC 20 kb)
Additional file 3:
**Weekly session themes.** (DOC 20 kb)
Additional file 4:
**A sample list of activities for session 1.** (DOC 20 kb)
Additional file 5:
**Diagram describing flow of patients through study.** (DOC 32 kb)
