# Supplementary material for: Participants’ perspectives on mindfulness-based cognitive therapy for inflammatory bowel disease: a qualitative study nested within a pilot randomised controlled trial
Source: Pilot Feasibility Stud. 2016 Jan 19;2:3. doi: 10.1186/s40814-015-0041-z (PMC5153874; doi:10.1186/s40814-015-0041-z)
Supplement: Additional file 1: — Topic guide schedule for the facilitator with preamble. (DOC 24 kb) [file 40814_2015_41_MOESM1_ESM.docx]

**Introduction**

Thank you for coming

Intro to the study (remind participants of the study aims). We are here today to try to explore further your views and thoughts on:

- evaluation of the MBCT program.

Outlining confidentiality and anonymity

Reminding that the group will be recorded

Outline group rules:

- Respecting other peoples’ views
- Not talking over each other
- Letting people talk and listen to them

Expectations of focus group and opportunity for participants to ask questions.

Any questions about study following from information sheet?

Interview/focus group will be informal, feel free to stop at any time.

Duration – no longer than 1 hour.

Assurance re: anonymity. No records will be kept with name on.

Any publications will be made anonymous and not identifiable to an individual or place. Verbal consent for interview and recording in addition to pre-attained written consent.

Microphone and battery check – START RECORDING

Ask everyone to introduce themselves starting with yourself.

*Expectations*

Tell us about what your expectations were regarding the self-help program if any?

Were your expectations met/ unmet in any way? And How?

*Length and difficulty of program*

Tell us about the length of eight weeks for the program, how acceptable was that for you?

Please tell us if there were specific parts of the program you found difficult to follow.

*Potential benefits*

Please tell us how you think the program benefited you.

What are your thoughts about the techniques you learned in the program

If you think you might continue to use some of the techniques, which ones they might be?

*Barriers to attending*

Can you please tell us what you think were barriers to attendance if any?

*What did you enjoy?*

Can you tell us if there which parts of the program did you most enjoy if any?

*Availability of MBCT program*

What are your thoughts about availability of MBCT

Can you tell us your thoughts if you think this program should be made available to IBD patients through NHS?
