# Supplementary material for: Participants’ perspectives on mindfulness-based cognitive therapy for inflammatory bowel disease: a qualitative study nested within a pilot randomised controlled trial
Source: Pilot Feasibility Stud. 2016 Jan 19;2:3. doi: 10.1186/s40814-015-0041-z (PMC5153874; doi:10.1186/s40814-015-0041-z)
Supplement: Additional file 2: — Free text survey questions. (DOC 20 kb) [file 40814_2015_41_MOESM2_ESM.docx]

*Expectations*

Did you have any expectations regarding the self-help program?

If yes, what were they?

Were your expectations met/ unmet in any way? And How?

*Length and difficulty of program*

Was the length of eight weeks for the program acceptable?

Did you find the program difficult to follow?

Please tell us which specific parts of the program you found difficult to follow.

*Potential benefits*

Do you think this program has brought any benefit to you?

Please tell us how you think the program benefited you.

Do you think you will continue to use some of the techniques you learned in the program?

*Barriers to attending*

Were there any barriers to attending the program?

Can you please tell us what you think were barriers to attendance?

*What did you enjoy?*

Did you enjoy the program?

Which parts did you most enjoy?

*Availability of MBCT program*

Do you think this program should be made available to IBD patients through NHS?
