# Supplementary material for: Participants’ perspectives on mindfulness-based cognitive therapy for inflammatory bowel disease: a qualitative study nested within a pilot randomised controlled trial
Source: Pilot Feasibility Stud. 2016 Jan 19;2:3. doi: 10.1186/s40814-015-0041-z (PMC5153874; doi:10.1186/s40814-015-0041-z)
Supplement: Additional file 3: — Weekly session themes. (DOC 20 kb) [file 40814_2015_41_MOESM3_ESM.docx]

**Weekly session themes**

Week 1: Awareness and automatic pilot

Week 2: Living in Our Heads

Week 3: Gathering the Scattered Mind

Week 4: Recognizing Aversion

Week 5: Allowing/Letting Be

Week 6: Thoughts Are Not Facts

Week 7: How Can I Best Take Care of Myself?

Week 8: Maintaining and Extending New Learning
