# Supplementary material for: Participants’ perspectives on mindfulness-based cognitive therapy for inflammatory bowel disease: a qualitative study nested within a pilot randomised controlled trial
Source: Pilot Feasibility Stud. 2016 Jan 19;2:3. doi: 10.1186/s40814-015-0041-z (PMC5153874; doi:10.1186/s40814-015-0041-z)
Supplement: Additional file 5: — Diagram describing flow of patients through study. (DOC 32 kb) [file 40814_2015_41_MOESM5_ESM.doc]

**Diagram describing flow of patients through study**

Randomized (n=44) (pilot RCT)

Allocated to MBCT group (n=22)

Allocated to waitlist control group (n= 22)

Lost to follow-up (n=10)

Lost to follow-up (n=10)

Eligible to participate in qualitative study (n=24)

Returned survey and participated in focus groups (n=18)
